# Exposure to salient, dynamic sensory stimuli during development increases distractibility in adulthood

**DOI:** 10.1038/srep21129

**Published:** 2016-02-17

**Authors:** Itay Hadas, Ram Gal, Lihi Bokovza, Nachshon Meiran, David Feifel, Abraham Zangen

**Affiliations:** 1 Department of Life Sciences and the Zlotowski Centre for Neuroscience, Ben-Gurion University of the Negev, Beer-Sheva, Israel; 2 Department of Psychology and the Zlotowski Centre for Neuroscience, Ben-Gurion University of the Negev, Beer-Sheva, Israel; 3 Department of Psychiatry and Neurosciences Program, University of California, San Diego, USA

## Abstract

It has been suggested that excessive exposure of children to the dynamic and highly salient audio-visual stimuli conveyed by electronic media may induce attention-related deficits in adulthood. This study was designed to evaluate this hypothesis in a controlled animal model setup. Building on their natural responsiveness to odors, we exposed juvenile rats for 1 h daily to a dynamic series of interchanging, highly salient odors, while controls were exposed to a non-changing mixture of these odors. Upon reaching adulthood, we tested the attentional capacity of the rats and measured their brain-derived neurotrophic factor (BDNF) levels as a proxy of neuronal plasticity. As compared with controls, rats exposed to the dynamic stimulation showed no attentional deficits under baseline task conditions, but their performance was dramatically impaired when an auditory distractor was introduced in the task. In addition, BDNF levels in the dorsal striatum of these rats were significantly increased relative to controls. These findings provide first empirical evidence that a continuous exposure to dynamic, highly salient stimuli has long-term effects on attentional functions later in life, and that these effects may have neural correlates in the dorsal striatum.

Several observational studies have correlated television viewing in early (“sensitive”) life periods with long-lasting deficits in various cognitive functions^1^,^2^. One function that appears to be particularly sensitive to the highly salient and dynamic stimuli conveyed by electronic media^1^—which today include not only television but also smartphones, computers, and other ubiquitous forms of electronic media—is attention and distractibility (for a recent review, see^3^). Because children today are exposed to electronic media at younger ages and for longer durations than in past decades^4^, and with the dramatic increase in the rates of ADHD over the past two decades^5^, several guidelines for limiting ‘screen time’ in children have been suggested^6^. Nevertheless, due to obvious practical and ethical considerations, the correlation between early exposure to electronic media and attentional deficits later in life cannot be tested empirically in humans. Thus, establishing a contributory role to the stimuli associated with electronic media in attentional deficits is today highly relevant.

In a seminal attempt to model the prolonged exposure of children to electronic media-like sensory stimulation, Christakis and colleagues^7^ exposed juvenile mice to flickering colored lights and to audio from the cartoon channel for 42 days. Consistent with the correlational observations in humans, the authors found that such exposure increased mice activity, decreased their anxiety, and impaired their short-term memory and spatial learning. These important findings called for further investigations of the causal connections between early life exposure to stimuli associated with electronic media and behavioral deficits later in life. For instance, attentional functions have not been directly tested in the study by Christakis *et al.* and the audio-visual stimulation, although possessing good face validity for screen-viewing in humans, introduces an ethologically discordant (and possibly aversive) feature into the animal model, as humans are primarily visual whereas rodents are primarily olfactory^8^.

In the current study, we performed a controlled experiment to directly investigate how attentional functions in adulthood are affected by exposing rats to dynamic and highly salient sensory stimuli during their early-life development. To measure attentional functions in the adult rats, we used the 5-Choice Serial Reaction Time Task (5-CSRTT) – a series of highly specific and widely used tests, which employ predominantly visual cues (with an auditory component in one of the tests; see below) and a quantitative and precise computerized touchscreen setup to measure various constructs of attention in rodents^9^. We used olfaction as the modality of sensory stimulation in the developing rats, for two main reasons: first, we consider olfaction to be an ethological analogue of the audio-visual stimuli associated with electronic media in children; and second, we aimed to avoid a direct interference with the development of the visual and auditory systems, which the 5-CSRTT uses to assess attentional functions. We thus exposed juvenile rats to a series of 12 distinct odors, which were changed by an experimenter every five minutes; we termed this procedure ‘developmental dynamic salient stimulation’ (DDSS) and maintained it, on a daily basis, for five weeks – a period roughly corresponding to pre-puberty and adolescence in a rat. The control group was exposed to the same odors but in a non-attention grabbing manner (see below), namely, as a static, non-changing mixture.

Under the hypothesis that a continuous and excessive exposure to attention-grabbing stimuli during development impairs various constructs of attention in adulthood^1^,^2^,^3^, we tested the attentional capabilities of adult DDSS and control rats in the 5-CSRTT paradigm. The task comprised several consecutive phases, including a pre-task exploration session, an acquisition period (in which the rat learns the task), a ‘baseline task’ phase, and a distractibility test (in which a white noise distractor is introduced in the regular task to test distraction filtering capability) (Fig. 1). In addition, we hypothesized that the predicted attention-related deficits in adult DDSS rats involves regional alterations in brain plasticity. To begin testing this hypothesis, we extracted the brains of DDSS and control rats following the DDSS and 5-CSRTT paradigms and analyzed the levels of brain-derived neurotrophic factor (BDNF), which was proven to be a stable and reliable proxy for brain plasticity^10^. We were primarily interested in potential BDNF alterations in the striatum, as several lines of converging evidence implicate this brain region in the cognitive abnormalities associated with attention-related deficits and, in humans, specifically in ADHD^11^,^12^,^13^. For instance, BDNF polymorphism in human adolescents has been associated with reward-related striatal functioning^12^, and the striatum has been shown (both in humans and in animal models) to play a crucial role in various executive and attentional functions, including in the regulation of selective attention^14^,^15^,^16^,^17^ and sustained attention^18^. Moreover, in rats, dorsomedial striatum deficits have been correlated with attentional deficits in the 5-CSRTT^19^, the task used in the present study, and dorsal striatum BDNF levels have been correlated with performance in a strategy set-shifting task (which taps executive functioning)^20^ and in other tasks that require working memory^21^. As an internal control, we also measured BDNF in the amygdala to identify possible stress- or fear-related confounds that may have resulted from the DDSS protocol^22^,^23^, and in the piriform cortex – a crucial region in olfactory processing^24^ – to identify possible long-term sensory-related alterations due to the chronic olfactory manipulation applied in the present study.

## Methods

### Animals

Male Sprague–Dawley rats (Harlan Laboratories, Rehovot, Israel) were housed in groups of three per cage on a reversed 12 h light/dark schedule (lights off at 10:00 AM). Food and water were available *ad libitum*. All experimental and handling protocols were approved by and conducted according to the regulations of the Institutional Animal Care and Use Committee (IACUC) of Ben-Gurion University of the Negev (authorization number: IL-51-08-2013), which are in complete accordance with the NIH guidelines for care and use of laboratory animals.

### Study design

**Figure 1:**
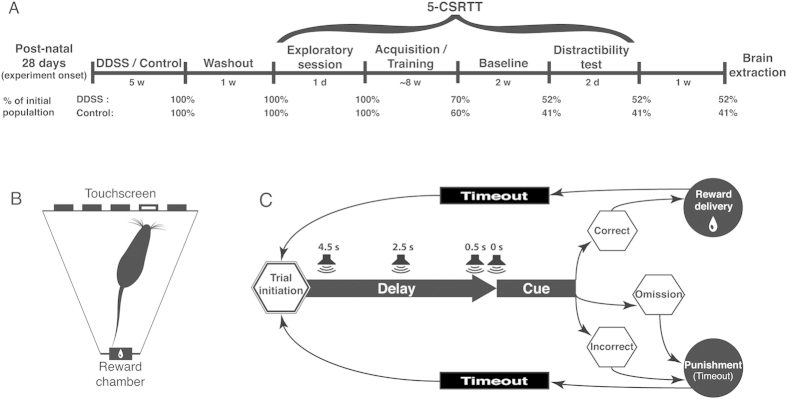
Study design. (**A**) Experimental timeline (schematic drawing, not to scale) and the percentage of rats reaching the predefined performance criteria at each stage of the 5-CSRTT. Initial sample sizes are n = 23 for DDSS rats and n = 17 for control rats. (**B**) Schematic drawing of the 5-CSRTT apparatus. A rat is shown facing a visual cue (white bar) displayed in one of the five apertures of the touchscreen. (**C**) The 5-CSRTT procedure. The trial is initiated by head-entry into the reward chamber, which triggers a 5-s delay followed by presentation of the visual cue light. Rats that nose-poke the aperture in which the visual cue is presented (“correct” response) are rewarded with sucrose solution delivered via a pump to the reward chamber, and can reinitiate the trial after a 5-s timeout. Rats that nose-poke another aperture (“incorrect” response) or that fail to respond to the visual cue altogether (“omission”) are punished by an extra 5-s timeout before being able to initiate the next trial. Rats that nose-poke any of the apertures before a cue is displayed (‘premature response’) are neither rewarded nor punished. The loudspeaker icons refer to the distractibility test only and represent the different possible time points during the delay period in which a white-noise distractor was interpolated. The distractor is interpolated randomly at different latencies (4.5, 2.5, 0.5, or 0 s) prior to displaying the visual cue.

The experimental timeline is depicted in Fig. 1A. Methodological details can be found in Supplementary Methods online, and are described here briefly.

#### Sensory stimulation

Three weeks old, post-weaned rats were exposed to 12 salient odorants (see Supplementary Table S1 online) for 1 h every weekday for a period of five weeks (p28–p63). Rats in the DDSS group were exposed to the odorants in a dynamic sequence, namely, by placing directly on top of their home cage an Eppendorf tube containing one odorant at a time (5 min per odorant, order of presentation determined randomly). Observations by an experienced experimenter indicated that the DDSS rats investigated the odorant tubes rigorously and continuously throughout the 1-h exposure sessions. Rats in the control group were exposed to a non-changing mixture of the same odorants; initially, we placed this mixture on top of their home cage (similar to DDSS rats), but preliminary observations indicated that such a setup considerably grabbed their attention and induced repeated exploratory approaches to the tube. These approaches presumably resulted in the rats perceiving dynamic intensities of the mixture of odors throughout the session – a ‘dynamic’ component of stimulation that we wanted to avoid in control rats. Hence, to decrease the dynamic attention-grabbing effect of the control stimulus altogether, we chose to distance the mixture of odors from these rats and placed the mixture of odors 2 m away from their home cage. Indeed, unlike DDSS rats, the control rats in this setup remained relatively inactive during the session, occasionally sniffing the constant and remote odors but not actively and dynamically interacting with their source.

#### 5-CSRTT

Following the 5-week stimulation period and another 1-week ‘washout’ period, all rats were submitted every weekday to the 5-CSRTT (Fig. 1B). The apparatus is a trapezoid chamber, in which the wider panel contains five apertures that the rat can nose-poke to establish contact with a touchscreen placed behind. The narrow panel houses a ‘reward chamber’ with a small dip in the floor, to which a pump can deliver the reinforcer (150 μl of a 10% sucrose solution). The design of a basic 5-CSRTT trial is shown in Fig. 1C and is detailed in the Supplementary Methods online. Briefly, a rat initiates a trial by head-entry into the reward chamber, triggering a 5-s delay followed by a visual (light) cue displayed in one of the five apertures on the front panel. Nose-poking the aperture associated with the cue light automatically delivers the reinforcer. The duration of cue display depends on task conditions. The session is terminated after 60 trials or after 60 min, whichever comes first.

The 5-CSRTT training procedure begins with a 30-min *exploratory session*, during which the rat is allowed to freely explore the apparatus. A sucrose solution is delivered non-contingently to the reward chamber prior to session onset to associate this chamber with the reward. The average time to poke the reward chamber for the first time and the pattern of exploration during the session are monitored. The following *acquisition phase* includes a one-week “conditioning” stage, in which the rat learns to nose-poke the apertures to receive a reward, followed by a “training” stage, in which the rat learns the basic task. In the training stage, a rat that meets all predefined performance criteria (PPC)—namely, at least 80% accuracy, a maximum of 20% omissions, and at least 50 completed trials per session—at the end of any individual session begins the next session with a shorter cue display duration (60 s, 30 s, 20 s, 10 s, or 5 s). A rat that does not fully meet all PPC continues to the next session without changing the cue display duration. A rat that meets all PPC for three consecutive sessions under the shortest (5 s) cue display condition is considered to have met the “baseline criteria” for the 5-CSRTT procedure and advances to the baseline phase. In the *baseline phase,* rats should maintain PPC under the 5 s cue display condition in a stable manner for 10 sessions. Rats that fail to meet these criteria for three consecutive days are excluded from further training or testing. Following the baseline phase, a *distractibility test* is conducted, in which a white noise distractor is interpolated at different latencies (4.5 s, 2.5 s, 0.5 s, 0 s, or none; determined randomly) throughout the delay period between trial initiation and cue display onset (loudspeaker icon in Fig. 1C). The results of two sessions performed in two successive days are averaged for each rat.

To minimize stress, all rats were allowed *ad libitum* access to food and water throughout the experimental period. Performance in the 5-CSRTT was motivated only by sugar and the number of rats meeting the PPC declined continuously as the experiment progressed (Fig. 1A). The number of rats reaching the PPC in each phase was not significantly different between DDSS and control rats (χ^2^ = 0.67, df = 11, p > 0.99).

The following behavioral parameters are extracted during the 5-CSRTT procedure: (i) the probability of correct responses; (ii) the probability of incorrect responses; (iii) the response accuracy [number of correct responses/(number of correct + incorrect responses) × 100]; (iv) the probability of omissions; (v) the probability of premature responses, interpreted as measurement for impulsivity (see, e.g.,^25^); and (vi) the correct-response reaction times (RTs). For the latter, the intra-individual distribution of the RTs of the correct responses were also analyzed by fitting the data with an ex-Gaussian probability density function^26^ – a well-established model of RT distributions, which is widely used to measure aspects of intra-individual RT variability^18^,^26^,^27^. Its parameters, calculated for each rat, are μ (corresponding to the mean of normal Gaussian component); σ (corresponding to the standard deviation of the normal Gaussian component); and τ (corresponding to the mean of the exponential component).

### BDNF measurements

Detailed protocols of the procedures can be found in the Supplementary Methods online. Briefly, brains were extracted one week after the last behavioral testing and bilateral tissue punches were obtained from the striatum (divided to dorsal and ventral striatum; dSTR and vSTR, respectively), basolateral amygdala (BLA), and piriform (Pir) cortex. Following protein extraction, samples were subjected to sandwich BDNF ELISA.

### Statistical analyses

In the exploratory session, the average latency to poke the reward chamber was compared between DDSS and control rats with a t-test. The exploration pattern during the active exploration phase (first 15 min of the session) was compared with a 2-way ANOVA, wherein Group (DDSS/control) and Location (touchscreen apertures/reward chamber) were the independent variables. In the acquisition phase, the behavior of DDSS and control rats was compared with a repeated-measures ANOVA, wherein Group (DDSS/control) and Cue Duration (60/30/20/10/5 s) were the independent variables and the number of sessions required to reach the predefined set of criteria was the dependent variable. In the baseline phase and in the distractibility test, the different measured parameters were compared between DDSS and control rats with a two-way ANOVA, wherein Group (DDSS/control) and Condition (baseline/distractibility test) were the independent variables. For the ex-Gaussian fitting function of the RTs, the μ, σ, and τ values of DDSS and control rats were compared with a t-test. In the distractibility test, the average session duration and the average number of trials per session in DDSS and control rats were compared with a t-test, and the effect of distractor latency (namely, the duration between the onset of the distractor and the onset of the cue) on performance was compared with a repeated-measures ANOVA, wherein Group (DDSS/control) and Latency (no distractor/4.5 s/2.5 s/0.5 s/0 s) were the independent variables. To asses differences in BDNF levels in DDSS and control rats, protein concentrations in each brain region were normalized to total protein, logarithmically transformed, and compared with a t-test. In all statistical analyses, the α level required to determine statistical significance was 0.05. A Bonferroni post-hoc test was used to determine specific differences between groups/conditions in all ANOVA tests. All results are expressed as means ± SEM unless stated otherwise.

## Results

### Exploratory session

**Figure 2:**
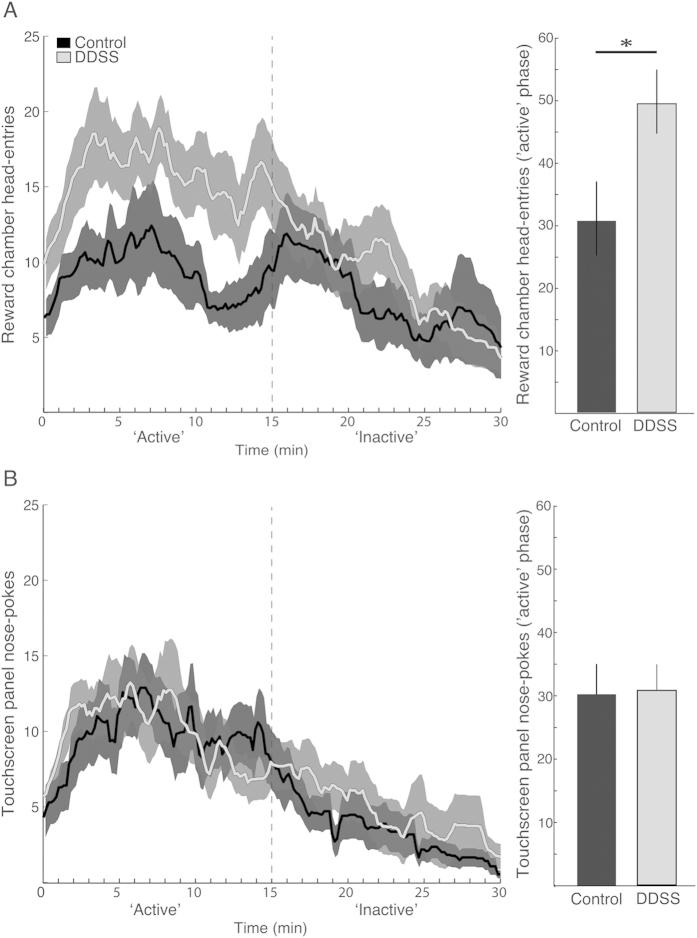
Exploratory session. The average number of head entries to the reward chamber (**A**) and the average number of nose-pokes in the touchscreen panel apertures (**B**) are shown for control (black; n = 17) and DDSS rats (grey; n = 23) in 1-min bins throughout the 30-min exploratory session (left panels) and, for the active exploration phase, as an overall average ( + SEM). *p < 0.05.

During the first 15 min of the exploratory session, all rats explored the novel 5-CSRTT apparatus, including nose-poking the touchscreen panel apertures and penetrating the reward chamber with their heads. The latency to discover the sucrose solution inside the reward chamber did not differ between DDSS and control rats (6.3 ± 2.0 min and 5.9 ± 1.6 min from the beginning of the session, respectively; p = 0.86, t-test). During the more active exploration phase of the session (first 15 min of the session; Fig. 2), a significant interaction was found between Group and Location (F(1, 38) = 5.01, p = 0.03; two-way ANOVA), wherein DDSS rats explored the reward chamber—but not the touchscreen apertures—more than control rats did (p = 0.04, Bonferroni post-hoc test; Fig. 2).

### Acquisition phase

**Figure 3:**
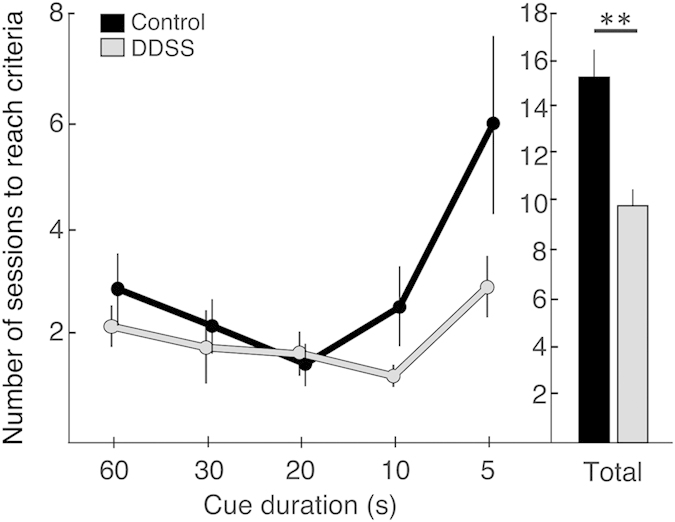
Acquisition phase. The number of sessions required to meet the predefined set of performance criteria is shown for DDSS rats (n = 16) and for control rats (n = 10) in each test condition (line graph) and as a cumulative total (bar graph). Values are mean ± SEM. The asterisks indicate a main effect for Group (p < 0.01) in the total number of sessions required to reach criteria.

The total number of sessions required to reach the PPC at the shortest (5-s) cue duration condition was significantly different between groups (F(1, 23) = 5.26, p = 0.03; repeated measures ANOVA), such that DDSS rats required fewer sessions overall to reach the PPC (Fig. 3). Although the difference between DDSS and control rats appeared to be more prominent in shorter cue durations (namely, in the 10 s and 5 s cue durations; Fig. 3), no significant Group × Cue duration interaction was found, and Bonferroni post-hoc tests did not indicate significant differences between groups with respect to cue duration.

### Baseline phase and the distractibility test

**Figure 4:**
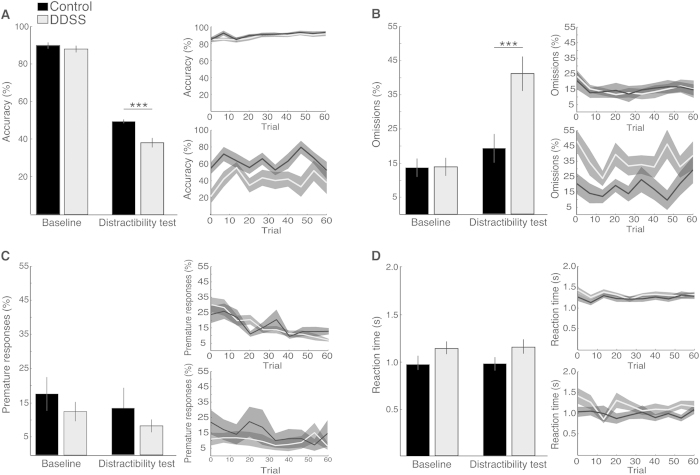
Performance in the baseline phase and in the distractibility test. The percentage of accurate responses (**A**), percentage of omissions (**B**), percentage of premature responses (**C**), and correct response reaction time (**D)** are shown for DDSS rats (grey, n = 12) and for control rats (black, n = 7) in the baseline phase and in the distractibility test. For each measured parameter in (**A–D**), the bar graphs show group averages ( + SEM) and the line graphs show session dynamics in the baseline phase (top panels) and in the distractibility test (bottom panels). For brevity, only group differences are shown (***p < 0.001), whereas differences between the baseline and the distractibility test are detailed in the text.

**Figure 5:**
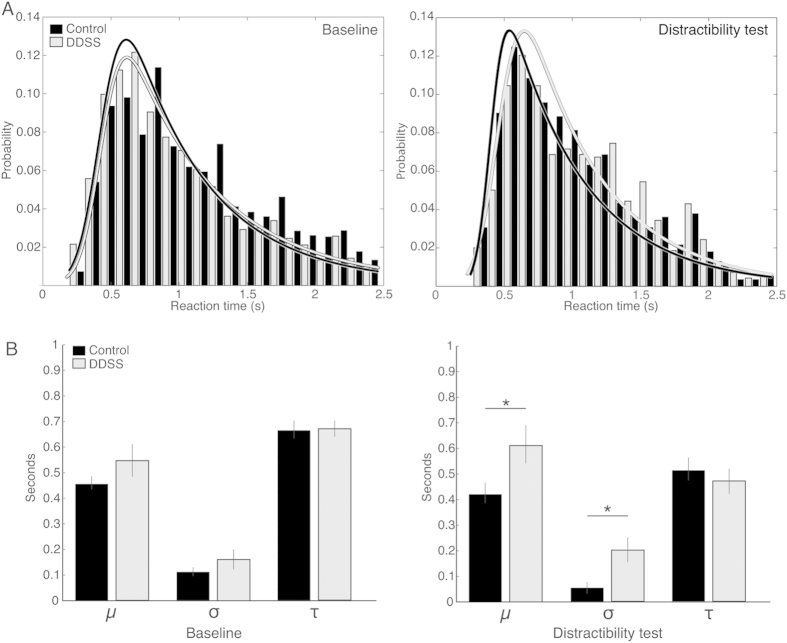
Intra-individual differences in the correct response reaction time. (**A**) Distribution histogram of the correct response reaction times of DDSS rats (grey; n = 12) and control rats (black, n = 7) in the baseline phase (left) and in the distractibility test (right). To illustrate the differences between the groups, the bars for each group indicate the cumulative averages, pooled from all trials and from all rats in the group. The line graphs represent the ex-Gaussian fit to these data. (**B**) Analyses of the ex-Gaussian probability density function of the correct response reaction times of individual rats. Values are mean ± SEM. *p < 0.05 (t-test between the groups).

A significant main effect was found for Condition (baseline phase versus distractibility test) in all examined parameters (Fig. 4), including response accuracy (F(1, 17) = 705.45, p = 0.00), probability of omissions (F(1, 17) = 1.19, p = 0.003), probability of premature responses (F(1, 17) = 7.66, p = 0.013), and RT (F(1, 17) = 14.68, p = 0.001). These results were expected and presumably indicate that the task requirements during the distractibility test were more difficult than those during the baseline phase for all rats (irrespective of the group). Importantly, a significant interaction was found between Group and Condition in response accuracy (F(1, 17) = 8.03, p = 0.01) and in the probability of omissions (F(1, 17) = 4.64, p = 0.04), such that DDSS rats performed less well than control rats (Fig. 4). No significant differences between the groups were found in the average duration for completing the distractibility test (43 min in DDSS rats and 41 min in control rats; p = 0.66, t-test) or in the average number of trials performed per session (54 in DDSS rats and 58 in control rats; p = 0.71, t-test) (data not shown). Analyses of the intra-individual variability in the RTs with an ex-Gaussian probability density function (Fig. 5) revealed no differences between the groups in the baseline phase, but, in the distractibility test, a significantly higher μ (p = 0.049) and σ (p = 0.026) were found in DDSS rats than in control rats.

**Figure 6:**
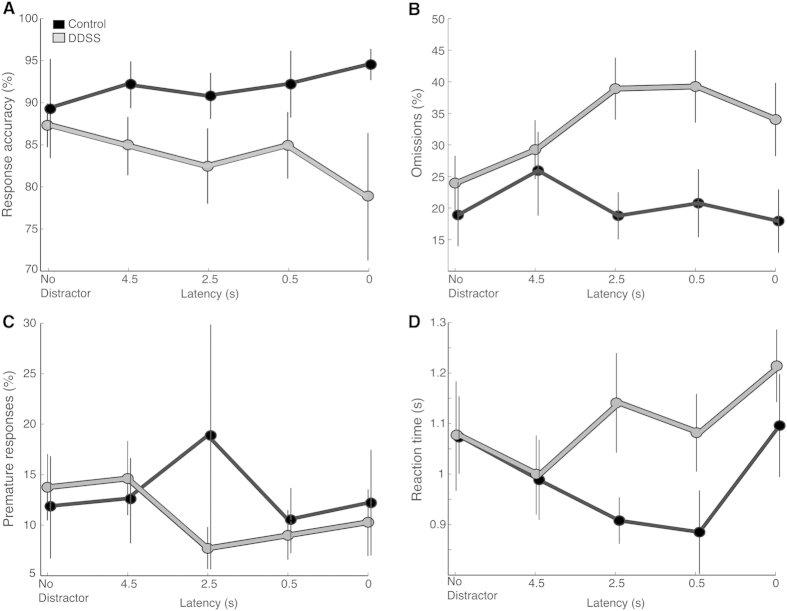
The effect of distractor-to-stimulus latency during the distractibility test. Performance of DDSS rats (grey, n = 12) and control rats (black, n = 7) is shown with respect to the different latencies between the onset of the distractor and the onset of the cue. Values are mean ± SEM of two sessions.

An in-depth analysis of the rats’ performance during the distractibility test (Fig. 6) revealed a significant main effect for Latency (i.e., the duration between the onset of the distractor and the onset of the cue) in the RTs (F(4, 64) = 3.32, p < 0.02) and a significant Group × Latency interaction in the probability of omissions (F(4, 68) = 3.28, p = 0.01, repeated-measures ANOVA). However, Bonferroni post-hoc tests did not find significant differences between the groups with respect to a specific latency in any of the measured parameters.

### BDNF measurements

**Figure 7:**
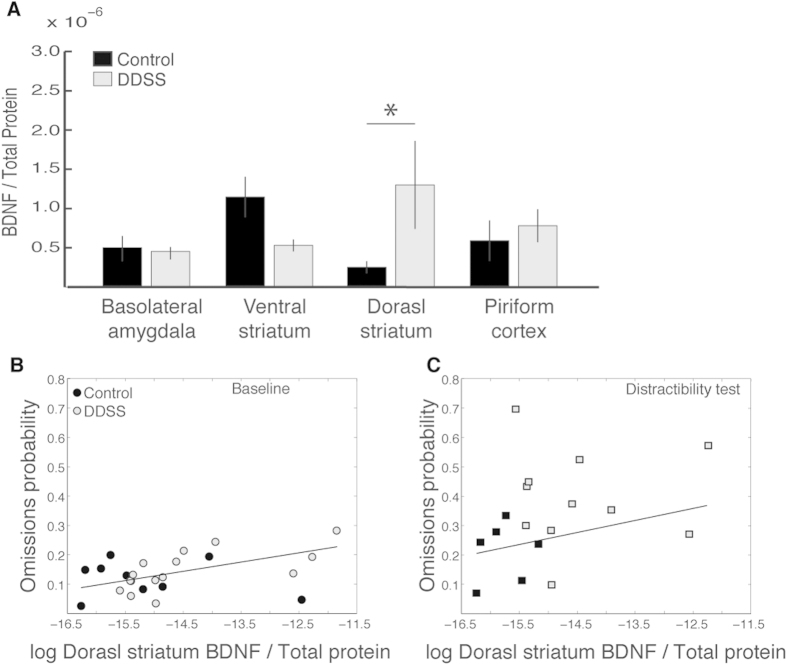
BDNF ELISA. (**A**) BDNF levels in attention-related brain structures (normalized to total protein in the sample). Values are mean ± SEM of rats that underwent the distractibility test (n = 12 DDSS rats and n = 7 control rats). *p < 0.05 (t-test on logarithmically transformed values). (**B,C**) Pearson correlations between BDNF levels in the striatum (logarithmic scale) and the probability of omissions in the baseline phase (**B**; n = 14 DDSS rats and n = 9 control rats; r = 0.58, p = 0.003) and in the distractibility test (**C**; n = 12 DDSS rats and n = 6 control rats; r = 0.33, p = 0.18). Grey data points indicate DDSS rats and black data points indicate control rats.

As a proxy for plasticity, BDNF levels in different brain structures associated with attention were measured one week following the distractibility test (Fig. 7A). BDNF levels in the dorsal striatum were significantly higher (p = 0.02; t-test) in DDSS rats than in control rats, whereas no differences were found in any of the other examined regions. BDNF levels in the dorsal striatum were also correlated positively with the percentage of omissions in the baseline phase (r = 0.58, p = 0.003; Fig. 7B) and in the distractibility test (a positive but not statistically significant correlation; r = 0.33, p = 0.18; Fig. 7C).

## Discussion

The mechanisms that drive population heterogeneity in attentional capabilities are not yet entirely clear^28^; whereas twin studies show that executive functions are near 100% heritable, they do not take into account the interaction between genes and the environment^29^,^30^. For instance, numerous studies have shown that attentional functions are influenced by genetic factors^29^,^31^,^32^, while other studies have shown that early-life environmental factors may play a significant role, too^33^,^34^,^35^,^36^,^37^. In the current study, we aimed to test whether prolonged exposure of juvenile rats to dynamic and highly salient stimuli will have long-term effects on attention. Notwithstanding the obvious differences between humans and animals, our study was motivated by the lack of direct, causal evidence for the common assumption that excessive exposure of young children to the dynamic and highly salient stimuli associated with electronic media may explain attentional impairments even years later, in adulthood. In addition, as several studies demonstrated that enriching the environment of young rodents with multisensory stimuli enhances—rather than hinders—brain development^38^,^39^, it appears that some aspects of the stimuli associated with electronic media may be beneficial to cognitive development, while others appear to be detrimental. Parsing these out will be critical for guiding the development of more salubrious forms of electronic media for children. The observation that rats exposed to the DDSS procedure in our study engaged the door-containing tubes continuously throughout the exposure period suggests that this paradigm was ethologically analogous to electronic media exposure in children, which is characterized by a high level of volitional engagement by children.

The results of this study prove empirically that a daily exposure of developing rats to dynamic, ethologically salient sensory stimuli for 5 weeks increases attentional distractibility in adulthood, indicating specific alterations in attentional processing. Notably, we found no attentional deficits in DDSS rats under baseline conditions; in fact, in the acquisition phase of the task, DDSS rats needed fewer sessions to acquire the task and reach the predefined set of performance criteria. Conversely, these rats performed significantly less well than control rats in the distractibility test. Because the 5-CSRTT relies on the visual and auditory modalities, whereas the DDSS protocol is predominantly olfactory, we believe that the attentional changes in rats exposed to DDSS did not stem from bottom-up sensory alterations; rather, they appear to be embedded in higher associative brain regions. Indeed, we found higher BDNF levels in the dorsal striatum of DDSS rats, a region that is anatomically and functionally interconnected with higher associative brain regions, and that is known to play a role in cognitive functions^40^. In contrast BDNF levels in the piriform cortex were not different between the groups, suggesting that the observed differences between the groups did not result from critical developmental alterations in sensory systems (see ref. 24). Additional studies are required to elucidate the specific brain structures and neuromodulatory circuits (e.g., dopaminergic^41^ or cholinergic^42^ deficits) that mediate these effects.

The distractibility of DDSS rats may be explained in light of their progressive increase in omissions when the cue–distractor latency was decreased. This phenomenon, which was significantly more pronounced in DDSS rats, resembles ‘attentional blink’ and is theorized to stem from the depletion of higher cognitive resources by the distractor^43^. Additionally, the RTs and the intra-individual RT variability were significantly higher in DDSS rats than in control rats during the distractibility test, but not under baseline conditions. In humans, increased intra-individual variability in RTs is an established measure of abnormalities in executive functions and has been suggested to comprise a core symptom of attention-related psychiatric pathologies^27^. Unfortunately, however, studies that measure intra-individual variability in animal models are scarce (see, e.g.,^18^,^44^) and, to the best of our knowledge, no such study utilized the ex-Gaussian distribution analysis, which enables deducing the intra-individual variability in a manner insensitive to the mean RT^26^,^27^,^45^,^46^. Another possible explanation for the distractibility of DDSS rats is that the procedure resulted in an ‘anxiety trait’, such that the audio distractor could have stressed those rats more than controls. However, we consider this explanation unlikely, as (i) DDSS rats did not show any signs of increased stress in their baseline performance, e.g., their RTs were not significantly different from those of control rats^47^,^48^; and (ii) their amygdala BDNF levels were similar to those in control rats, suggesting that the developmental manipulation did not consolidate as a ‘fear memory'^49^ or resulted in a long-term ‘anxiety trait'^22^.

An unexpected finding was that, during the pre-task exploratory session, DDSS rats showed increased exploration of the “reward chamber”, wherein a sucrose solution was placed non-contingently prior to the initiation of the session. In contrast, the number of nose-pokes in the touchscreen panel apertures was similar in both groups, suggesting that DDSS rats are generally not hyperactive. Nevertheless, future studies should directly measure hyperactivity, as well as other behavioral constructs following the DDSS procedure. Given the demanding, time-consuming and long-term training required for the delicate 5CSRT task, such additional behavioral measures would best be performed on separate groups of animals to avoid stress and overload of demanding behavioral measures that could potentially affect each other. Early life experiences are known to dramatically influence brain structure and function to provide the organism with a unique opportunity to adapt to the specific environment to which it is exposed^50^. Accordingly, one explanation to the greater “reward chamber” (but not touchscreen) exploration observed in DDSS (relative to control) rats during the pre-task exploratory session is that the adult DDSS rats, who were continuously and repeatedly exposed to highly salient and dynamic exogenous stimuli as juveniles, assigned higher saliency values (i.e., allocated greater attentional resources) to exogenous stimuli that control rats rapidly adapted to. This phenomenon, termed ‘incentive salience attribution’^51^, corroborates with the increased sensitivity to incentives that was reported in children and adults with ADHD, and probably stems from abnormal [probably dopaminergic^52^] striatal activation in this population^53^,^54^,^55^. In line with this speculation, the better performance of DDSS rats in the acquisition phase—and, especially, in the shorter cue conditions, which required a higher degree of attention to the cue—may have resulted from higher saliency values that those rats assigned to the cues. The differences between the two groups may have dissipated during the baseline phase because the task became more habitual and required less attention being allocated to the cue; in a somewhat similar manner, humans with ADHD perform better under more demanding tasks^56^. Finally, assigning a higher saliency value to exogenous stimuli may also explain the impairment in performance of DDSS rats in the distractibility test, which involved the interpolation of a salient, unpredicted audio stimulus in the regular task.

As an initial step toward elucidating neurochemical correlates of behavioral alterations induced by DDSS, we measured BDNF levels as a proxy for plasticity-related, long-term mechanisms that may potentially alter attentional functions later in life. Increased BDNF levels has been documented in various brain regions (including striatum) of animals exposed to environmental enrichment^57^,^58^,^59^, and higher BDNF levels in the plasma has been correlated with ADHD-related symptoms in children^60^. In the present study, we found BDNF levels to be increased in the dorsal striatum of rats exposed to DDSS, a brain region implicated in sensorimotor integration and in various cognitive functions, including selective attention (and its derivative – distractibility)^14^,^15^,^16^,^17^ and sustained attention^18^. In rats, Rogers and colleagues^19^ found that lesions of the dorsomedial striatum decreased response accuracy and increased RTs in the 5-CSRTT, and D’Amore and colleagues^20^ reported that administering BDNF directly to the dorsal striatum reduced the number of sessions required to reach criteria, increased RTs, and increased the number of omissions in a strategy set-shifting task, which taps executive functioning. The cognitive deficits of those rats are strikingly similar to those observed in DDSS rats in the distractibility test in our study. Moreover, rats with decreased striatal BDNF levels show impaired performance in tasks requiring working memory^21^, and the increased BDNF levels in the dorsal striatum of DDSS rats may thus explain, at least in part, their faster task acquisition. Finally, BDNF polymorphism in human adolescents has been associated with reward-related striatal functioning^12^, which may be reminiscent of the increased reward chamber exploration exhibited by DDSS rats during the initial session.

To conclude, the results of this prospective animal study provide empirical evidence for the claim that early exposure to electronic media in children plays a causal role in distractibility that these children exhibit later in life. Our data suggest that the dynamic presentation of highly salient stimuli is one feature of current-day electronic media that may produce negative effects on the development of attention. Changes in BDNF (a proxy for brain plasticity) in the dorsal striatum may underlie these long-term behavioral changes, although this assumption should be tested more directly in future studies. Finally, the DDSS method reported here appears to be a useful, ethologically relevant, animal model for studying the effects of early exposure to electronic media in developing humans. Future studies, possibly utilizing electrophysiological or pharmacological approaches, can use this model to further explore the mechanisms underlying the reported phenomena.

## Additional Information

**How to cite this article**: Hadas, I. *et al.* Exposure to salient, dynamic sensory stimuli during development increases distractibility in adulthood. *Sci. Rep.* **6**, 21129; doi: 10.1038/srep21129 (2016).

## Supplementary Material

Supplementary Information
