# Supplementary material for: Exposure to salient, dynamic sensory stimuli during development increases distractibility in adulthood
Source: Sci Rep. 2016 Feb 17;6:21129. doi: 10.1038/srep21129 (PMC4756326; doi:10.1038/srep21129)
Supplement: Supplementary Information [file srep21129-s1.doc]

## Exposure to salient, dynamic sensory stimuli during development increases distractibility in adulthood

Itay Hadas, Ram Gal, Lihi Bokovza, Nachshon Meiran, David Feifel, and Abraham Zangen

**Supplementary Methods**

## Developmental dynamic salient stimulation

Post-weaned rats were subjected to daily 1-h 'exposure sessions' for five weeks (p28–p63). All exposure sessions were conducted during the dark phase (1:00–4:00 PM) in a small room with red fluorescent lighting. In the DDSS group, an Eppendorf tube containing 20 µl of the odorant (soaked in cotton) was placed on top of the home cage for 5 minutes, after which it was quickly replaced with another tube containing a different odorant. All odorants (detailed in Supplementary Table 1) were purchased from Florasynth (Holon, Israel) and are used in the food industry. In each 'exposure session', 12 different odorants were presented sequentially to each rat in the DDSS group in a random order. In the control group, a mixture of the same 12 odors was placed approximately 2 m from the home cage; those rats were thus exposed to all odors but not in a sequential, dynamic manner. Both groups were similarly handled daily and habituated to the experimenter to reduce potential differences associated with general stress.

**The 5-CSRT task**

**Apparatus**

The 5-CSRTT was conducted in prebuilt trapezoid aluminum Bussey–Saksida touchscreen operant chambers for rats (Campden Instruments, Loughborough, UK), as depicted in Figure 1B. These chambers were placed on an aluminum mesh and were covered with a Plexiglass cover. The front panel of the chamber (i.e., its wider side) contained five 1-cm deep apertures that the rat could nose-poke to establish contact with a specialized touchscreen placed behind the panel. The rear (narrow) panel of the chamber contained a 'reward chamber'; a pump delivered the reinforcing sucrose solution into a small dip at the bottom of this chamber. A white LED, positioned above the 'reward chamber', was turned on in association with reward delivery. All operant chambers were kept in sound- and light-proof boxes equipped with house lights in the roof of the chamber, an audio loudspeaker (for delivery of the distractor during the distractibility test), and fans that provided a constant low-level white noise and airflow. The entire apparatus was operated and task-related behaviors were monitored through designated software (AbetII, Lafayette Instruments, Lafayette, IN, USA).

**Task**

The 5-CSRTT is a well-established paradigm for assessing various attention-related functions in rodents ^1^. The basic design of a 5-CSRTT trial is depicted in Figure 1C. Briefly, in each session, rats are placed individually in the apparatus and initiate a trial by head-poking the reward chamber, which triggers a 5-s delay followed by a visual (light) cue that is displayed in one of the five apertures on the front panel (the duration of cue display depends on task conditions). A 'correct response' is recorded if the rat responds to the visual cue by nose-poking the aperture in which the cue was displayed; an 'incorrect response' is recorded if the rat nose-pokes an aperture different from the 'correct' one; a 'premature response' is recorded if the rat nose-pokes any of the five apertures before the cue was displayed; an 'omission' is recorded if the rat fails to nose-poke any of the five apertures for a certain amount of time, which depends on task conditions, after the cue was displayed. In each trial, a 'correct response' rewards the rat with 150 µl of a 10% sucrose solution delivered to the reward chamber, whereas an 'incorrect response' or an 'omission' result in a 'failure' signal (turning on the house lights) followed by a 5-s timeout period, during which the rat cannot initiate a new trial. Premature responses are recorded but are not punished. Each session was concluded after 60 trials or after 60 min, whichever came first.

The software associated with the 5–CSRTT allowed us to extract numerous behavioral parameters for each phase of the paradigm, most importantly the probabilities of correct and incorrect responses, the response accuracy [number of correct responses / (number of correct + incorrect responses) × 100), the probability of omissions, the probability of premature responses (which are interpreted as measurement for impulsivity; ^2–6^), and, for the correct responses, the reaction times probability density function that is interpreted as individual animal attention functional consistency ^7–13^. The interpolation of the white-noise distractor at different time points during the delay (Figure 1C, and see below for details) allows assessing the distractibility of the rat ^1,14,15^.

*Exploratory session*

One week after the final DDSS (or control) exposure session, rats were placed individually in the 5-CSRTT apparatus for 30 min to explore and familiarize with the apparatus. A sucrose solution was placed in the reward chamber to familiarize the rats with this chamber and associate it with the reward solution. Although no task was conducted during the exploratory session, the average time to poke the reward chamber for the first time and the pattern of exploration during the session were monitored automatically, as in all other sessions.

*Acquisition phase*

This phase included a one-week "conditioning" stage, in which rats learned the basic task (touching the screen to receive a reward), followed by a "training" stage, in which the duration of displaying the visual cue was decreased gradually between sessions (60 s, 30 s, 20 s, 10 s, 5 s). The duration of the cue was decreased each time the rat reached a predefined set of performance criteria: at least 80% accuracy, a maximum of 20% omissions (calculated as the number of trials with no response / total number of trials × 100), and at least 50 completed trials per session. Thus, a rat that met all these criteria at the end of any individual session began the next session with a shorter duration of visual cue display, whereas a rat that did not fully meet these criteria continued to the next session without changing the duration of the cue display. A rat that met these criteria for three consecutive sessions under the shortest (5 s) cue display condition was considered to have met the "baseline performance criteria" for the 5-CSRTT, and continued to the baseline phase. Rats that did not meet those baseline criteria in a stable manner for two weeks were removed from the acquisition phase and were excluded from further training or testing.

*Baseline phase and distractibility test*

Following the acquisition phase, rats were maintained at baseline criteria for two weeks (10 sessions) to establish the baseline performance parameters of each group. Rats that did not maintain baseline criteria for 3 consecutive days were omitted from the experiment. Following the baseline phase, a distractibility test was conducted in which an audible white noise distractor was interpolated at different latencies (namely, 4.5 s, 2.5 s, 0.5 s, 0 s, or none) throughout the delay period between trial initiation and cue display onset (Figure 1C). The test was conducted in two successive days and the results of the two sessions were averaged for each rat.

In both the baseline phase and the distractibility test, we calculated the percentage of omissions, accuracy, premature responses, and the reaction time for correct responses, as described elsewhere ^1^. We also analyzed the intra-individual distribution of the reaction times of the correct responses by fitting the data with an ex-Gaussian probability density function available for Matlab ^10^. The ex-Gaussian distribution is a well-established model of reaction time distributions and is widely used to measure aspects of intra-individual reaction time variability ^7^. Its parameters are µ (corresponding to the mean of normal Gaussian component); σ (corresponding to the standard deviation of the normal Gaussian component); and τ (corresponding to the mean of the exponential component). These parameters were calculated for each rat. Notice that the reaction time distribution in Figure 5 is shown for illustrative purposes only and demonstrates cumulative data from all trials and from all rats, averaged for each group.

**BDNF analyses**

**Histology and tissue punches**

One week after the last behavioral testing, rats were sacrificed and their brains extracted and frozen in isopropanol for storage at –80 °C. Bilateral tissue punches were obtained from coronal sections generated by a manual cut within a cryostat (Leica CM 3050 S, Germany) maintained at –20°C, as previously described ^16,17^. Punches were extracted from the basolateral amygdala (BLA), nucleus accumbens (NAc), dorsal striatum (dSTR), and piriform (Pir) cortex. The location of punches was determined according to the anteroposterior position relative to bregma in the rat brain atlas ^18^: BLA: –2.5 mm to –3.5 mm; NAc: 2.5 mm to 0.5, dSTR: 2.5 mm to 0.5; Pir: –3.5 mm to –4.5 mm.

**Protein extraction**

Protein extraction was performed as described previously by ^19^. Brain tissue samples were weighed and homogenized in a cold extraction buffer and homogenates were then acidified with 0.1 M HCl (pH ~3.0), incubated at room temperature for 15 min, and neutralized with 0.1 M NaOH (pH ~7.6). Homogenates were then microfuged at 7000 g for 10 min, and supernatants were assayed with a BDNF ELISA.

**BDNF ELISA**

Sandwich ELISA was carried out as described previously ^19^ with slight modifications ^20^. Briefly, monoclonal mouse anti-human BDNF capture antibody (R&D Systems, Inc., Minneapolis, MN) was incubated overnight in 96-well flat-bottomed polystyrene plates. After incubation, the wells were washed and a blocking buffer was added to each well. After washing three times, homogenized brain samples (50 μl/ well) were added in duplicates. Positive (BDNF) and negative (Reagent Diluent) controls were included. After incubation and washing, mouse anti-human BDNF detection antibody was added, and the plates were incubated. After three washes, streptavidin conjugated to horseradish peroxidase was added and the plates were incubated in darkness. The substrate was then added and the color was allowed to develop for 20 min in darkness, after which the reaction was stopped with H_2_SO_4_. The plates were read at 450–550 nm by using a microplate reader (Thermo Fisher Scientific Oy, Ratastie, Vantaa, Finland).

**References**

1. Carli, M., Robbins, T. W., Evenden, J. L. & Everitt, B. J. Effects of lesions to ascending noradrenergic neurones on performance of a 5-choice serial reaction task in rats; implications for theories of dorsal noradrenergic bundle function based on selective attention and arousal. *Behav. Brain Res.* **9,** 361–380 (1983).

2. R. N. Cardinal, David R. Pennicott, C. Lakmali Sugathapala, Trevor W. Robbins & Barry J. Everitt. Impulsive Choice Induced in Rats by Lesions of the Nucleus Accumbens Core. *Science* **292,** 2499–2501 (2001).

3. Dalley, J. W. *et al.* Nucleus Accumbens D2/3 Receptors Predict Trait Impulsivity and Cocaine Reinforcement. *Science* **315,** 1267–1270 (2007).

4. Jupp, B. *et al.* Dopaminergic and GABA-ergic markers of impulsivity in rats: evidence for anatomical localisation in ventral striatum and prefrontal cortex. *Eur. J. Neurosci.* n/a–n/a (2013). doi:10.1111/ejn.12146

5. Eagle, D. M. & Robbins, T. W. Inhibitory Control in Rats Performing a Stop-Signal Reaction-Time Task: Effects of Lesions of the Medial Striatum and d-Amphetamine. *Behav. Neurosci.* **117,** 1302–1317 (2003).

6. Christakou, A., Robbins, T. W. & Everitt, B. J. Functional disconnection of a prefrontal cortical-dorsal striatal system disrupts choice reaction time performance: Implications for attentional function. *Behav. Neurosci.* **115,** 812–825 (2001).

7. Kofler, M. J. *et al.* Reaction time variability in ADHD: A meta-analytic review of 319 studies. *Clin. Psychol. Rev.* **33,** 795–811 (2013).

8. Schneider, T. *et al.* Prenatal exposure to nicotine impairs performance of the 5-choice serial reaction time task in adult rats. *Neuropsychopharmacol. Off. Publ. Am. Coll. Neuropsychopharmacol.* **36,** 1114–1125 (2011).

9. Castellanos, F. X. *et al.* Varieties of Attention-Deficit/Hyperactivity Disorder-Related Intra-Individual Variability. *Biol. Psychiatry* **57,** 1416–1423 (2005).

10. Lacouture, Y. & Cousineau, D. How to use MATLAB to fit the ex-Gaussian and other probability functions to a distribution of response times. *Tutor. Quant. Methods Psychol.* **4,** 35–45 (2008).

11. Swanson, J. *et al.* Attention deficit/hyperactivity disorder children with a 7-repeat allele of the dopamine receptor D4 gene have extreme behavior but normal performance on critical neuropsychological tests of attention. *Proc. Natl. Acad. Sci.* **97,** 4754–4759 (2000).

12. Loos, M. *et al.* Inhibitory control and response latency differences between C57BL/6J and DBA/2J mice in a Go/No-Go and 5-choice serial reaction time task and strain-specific responsivity to amphetamine. *Behav. Brain Res.* **214,** 216–224 (2010).

13. Cummins, T. D. R. *et al.* Alpha-2A adrenergic receptor gene variants are associated with increased intra-individual variability in response time. *Mol. Psychiatry* **19,** 1031–1036 (2014).

14. Cole, B. J. & Robbins, T. W. Effects of 6-hydroxydopamine lesions of the nucleus accumbens septi on performance of a 5-choice serial reaction time task in rats: Implications for theories of selective attention and arousal. *Behav. Brain Res.* **33,** 165–179 (1989).

15. Pezze, M.-A., Dalley, J. W. & Robbins, T. W. Differential roles of dopamine D1 and D2 receptors in the nucleus accumbens in attentional performance on the five-choice serial reaction time task. *Neuropsychopharmacol. Off. Publ. Am. Coll. Neuropsychopharmacol.* **32,** 273–283 (2007).

16. Taliaz, D., Nagaraj, V., Haramati, S., Chen, A. & Zangen, A. Altered Brain-Derived Neurotrophic Factor Expression in the Ventral Tegmental Area, but not in the Hippocampus, Is Essential for Antidepressant-Like Effects of Electroconvulsive Therapy. *Biol. Psychiatry* **74,** 305–312 (2013).

17. Gersner, R., Gal, R., Levit, O., Moshe, H. & Zangen, A. Inherited behaviors, BDNF expression and response to treatment in a novel multifactorial rat model for depression. *Int. J. Neuropsychopharmacol.* **17,** 945–955 (2014).

18. Paxinos, G. & Watson, C. *The rat brain in stereotaxic coordinates*. (Elsevier/Academic, 2009).

19. Baker-Herman, T. L. *et al.* BDNF is necessary and sufficient for spinal respiratory plasticity following intermittent hypoxia. *Nat. Neurosci.* **7,** 48–55 (2004).

20. Zilkha, N., Feigin, E., Barnea-Ygael, N. & Zangen, A. Induction of depressive-like effects by subchronic exposure to cocaine or heroin in laboratory rats. *J. Neurochem.* **130,** 575–582 (2014).

**Table S1**: Odorants used for the DDSS exposure (all purchased from Florasynth TM., Israel)

| **Odorant** | **Variant** | **CAT No.** |
| --- | --- | --- |
| **Strawberry** | 4011 | 100882 |
| **Milk condensed** | 3158/4 | 100232 |
| **Apricot** | 3000BZ | 111101 |
| **Mango** | 1836 | 109711 |
| **Grapefruit** | T.V.I | 100534 |
| **Pineapple (Ananas)** | 5722 | 100100 |
| **Banana** | A.G | 100201 |
| **Rum** | MNS | 100839 |
| **Grape** | VGP | 100156 |
| **Lemon fresh** |  | 100679 |
| **Orange** | 5168 | 100758 |
| **Hazelnut** | 7470 | 100605 |
| **Almond** | 2212 | 100118 |
| **Raspberry** | 8011 | 100833 |
| **Vanilla** | 101/A | 100892 |
| **Fruits of forest** |  | 10017 |
| **Honey** | 2150 | 100601 |
| **Mocca** | 631/404 | 100704 |
| **Shamenet arome** ('cream') |  | 100872 |
